# The effect of IL-13 and IL-13R130Q, a naturally occurring IL-13 polymorphism, on the gene expression of human airway smooth muscle cells

**DOI:** 10.1186/1465-9921-6-9

**Published:** 2005-01-20

**Authors:** Farhat Syed, Reynold A Panettieri, Omar Tliba, Chris Huang, Katherine Li, Michelle Bracht, Bernard Amegadzie, Don Griswold, Li Li, Yassine Amrani

**Affiliations:** 1 Centocor Inc., 200 Great Valley Parkway, Malvern PA 19355. USA; 2 Pulmonary Allergy and Critical Care Division, Department of Medicine, University of Pennsylvania, Room 848 BRBII/III, 421 Curie Boulevard, Philadelphia PA 19104. USA

## Abstract

**Background:**

Growing evidence shows that interleukin 13 (IL-13) may play an essential role in the development of airway inflammation and bronchial hyper-responsiveness (BHR), two defining features of asthma. Although the underlying mechanisms remain unknown, a number of reports have shown that IL-13 may exert its deleterious effects in asthma by directly acting on airway resident cells, including epithelial cells and airway smooth muscle cells. In this report, we hypothesize that IL-13 may participate in the pathogenesis of asthma by activating a set of "pro-asthmatic" genes in airway smooth muscle (ASM) cells.

**Methods:**

Microarray technology was used to study the modulation of gene expression of airway smooth muscle by IL-13 and IL-13R130Q. TaqMan™ Real Time PCR and flow cytometry was used to validate the gene array data.

**Results:**

IL-13 and the IL-13 polymorphism IL-13R130Q (Arg130Gln), recently associated with allergic asthma, seem to modulate the same set of genes, which encode many potentially interesting proteins including vascular cellular adhesion molecule (VCAM)-1, IL-13Rα2, Tenascin C and Histamine Receptor H1, that may be relevant for the pathogenesis of asthma.

**Conclusions:**

The data supports the hypothesis that gene modulation by IL-13 in ASM may be essential for the events leading to the development of allergic asthma.

## Background

Recent reports using murine models of allergic asthma have shown that the Th2 type cytokine IL-13 may play a critical role in the pathogenesis of asthma, either by regulating airway inflammation, mucus hyper-secretion or airway hyper-responsiveness [1-5]. Evidence suggests that the potential role of IL-13 in asthma may come from its aptitude to directly interact with airway resident cells, such as epithelial cells or airway smooth muscle (ASM) cells, as shown by the ability of IL-13 to stimulate a set of different pro-asthmatic genes including inflammatory cytokines such as thymus and activation-regulated chemokine (TARC), eotaxin, monocyte chemotactic protein-1 (MCP-1) as well as growth factors such as vascular endothelial growth factor (VEGF) and basic fibroblast growth factor (bFGF) [6-10]. The ability of IL-13 to modulate ASM responsiveness to G-protein coupled receptor (GPCR) agonists, either by increasing contractile agonist-evoked calcium responses [11], and/or by impairing ASM responsiveness to β2-adrenoceptor stimulation [6], may also explain, at least in part, the putative role of IL-13 in allergen-associated BHR reported in animal models [1-4]. Previous reports have shown that other cytokines such as tumor necrosis factor alpha (TNFα) or interleukin (IL)-1β, may also participate in airway hyper-responsiveness by modulating ASM responsiveness to contractile GPCR agonists [12-14]. These data strongly support the current concept that cytokine modulation of ASM, an effector cell thought to solely regulate bronchomotor tone [12], may play an important role in the development of airway inflammation and bronchial hyper-responsiveness, the two main features of asthma. The molecular mechanisms by which IL-13 induces "pro-asthmatic responses" in ASM have not been clearly established. Identifying the expression profile of "pro-asthmatic" genes activated by IL-13 in ASM cells may therefore provide new insight into the design of novel therapeutic approaches for asthma.

Using complementary molecular approaches, we investigated the effect of IL-13 on the transcription of "pro-asthmatic" genes in human airway smooth muscle cells (HASMC). The effect of IL-13 was compared to that of IL-13R130Q, a naturally occurring isoform resulting in a change from glutamine to arginine residues in the coding region that is associated with high serum IgE levels [15]. Interestingly, no report has yet investigated whether both IL-13 and IL-13R130Q share the same or have different biological activities. We found that both IL-13 and IL-13R130Q stimulate the same set of important genes that encode for proteins which may be clinically relevant for regulating airway hyper-responsiveness, airway inflammation and airway remodeling, key characteristics of asthma.

## Methods

### Cell Culture

Human tracheas were obtained from lung transplant donors, in accordance with procedures approved by the University of Pennsylvania Committee on Studies Involving Human Beings. A segment of trachea just proximal to the carina was removed under sterile conditions and the tracheal muscle was isolated. The muscle was then centrifuged and resuspended in 10 ml of buffer containing 0.2 mM CaCl_2_, 640 U/ml collagenase, 1 mg/ml soybean trypsin inhibitor and 10 U/ml elastase. Enzymatic dissociation of the tissue was performed for 90 min in a shaking water bath at 37°C. The cell suspension was filtered through 105 μm Nytex mesh, and the filtrate was washed with equal volumes of cold Ham's F12 medium (Gibco BRL Life Technologies, Grand Island, NY) supplemented with 10% FBS (HyClone, Logan, UT) 100 U/ml penicillin (Gibco), 0.1 mg/ml streptomycin (Gibco), and 2.5 μg/ml fungizone (Gibco). Aliquots of the cell suspension were plated at a density of 1.0 × 10^4 ^cells/cm^2^. The cells were cultured in Ham's F12 media supplemented with 10% FBS, 100 U/ml penicillin, 0.1 mg/ml streptomycin and this was replaced every 72 h. Human ASM cells in subculture during the second through to fifth cell passages were used because, during these cell passages, the cells retain native contractile protein expression, as demonstrated by immunocytochemical staining for smooth muscle actin and myosin [16]. Unless otherwise specified, all chemicals used in this study were purchased from Sigma/Aldrich (St. Louis, MO).

### RNA isolation

Total cellular RNA was isolated from IL-13 (50 ng/ml), IL-13R130Q (50 ng/ml) or control treated HASMC using the RNeasy mini kit (Qiagen, Inc. Valencia, CA) as per manufacturer's instructions. The IL-13 was purchased from R&D Systems (Minneapolis, MN) and the IL-13R130Q was generated in house at Centocor Inc. The quality and quantity of RNA was assessed using the Agilent 2100 Bioanalyzer (South Plainfield, New Jersey). Samples that demonstrated high quality (ratio of 28S rRNA and 18S rRNA is greater than 1.7) were submitted for microarray analysis.

### Microarray Processing

A complimentary DNA (cDNA) microarray, or DNA chip (Target B), containing a total of 8159 human gene cDNA clones from Research Genetics (IMAGE consortium, Huntsville, AL), Incyte Genomics (Santa Clara, CA) and internal sources was used in this study. All clones have been verified by DNA sequencing and are printed as 2 independent spots on a given chip. Duplicate chips were used for each RNA sample. Non-linear normalization between duplicate chips allowed each clone to be averaged to a single intensity value for each RNA sample.

RNA amplification, probe synthesis and labeling, cDNA chip hybridization and washing were performed as described previously [17]. Agilent Image Scanner was used to scan the cDNA chips (Agilent Technologies, Palo Alto, CA). Fluorescence intensity for each feature of the array was obtained by using ImaGene software (BioDiscovery, Los Angeles, CA).

### Microarray data analysis

In this study, fifty one-color cDNA microarrays were used to profile gene expression in human airway smooth muscle cells from 3 donors stimulated with IL-13, or its variant IL-13R130Q at 2 time points (6 hr and 18 hr). Untreated samples from the same group of donors were used as control. The samples being analyzed are listed in Table 1.

**Table 1:** Summary of number of samples from each donor and treatments

Time	Donor	Untreated	IL-13	IL-13R130Q
6 hr	Donor 1*	-	-	-
	Donor 2	1	2	2
	Donor 3	1	2	2
				
18 hr	Donor 1	1	2	2
	Donor 2	1	2	2
	Donor 3	1	2	2

*The samples from Donor 1 at the 6 hr time point were not included due to poor quality of RNA.

Purified cDNA probes were hybridized to two microarrays, each containing two spots for each cDNA. Raw intensity data from the cDNA arrays were first normalized within each sample. Linear normalization and then nonlinear normalization was performed within each sample. Outlier intensity data points (greater than 1.4 fold away from the median of replicate measurements) were identified and removed from the data sets. The average intensity was generated by calculating the arithmetic mean of nonoutlier intensity values. The averaged intensity for each clone was further normalized across all samples. Chip-to-chip normalization was performed by dividing the averaged intensity of each clone by the 50.0^th ^percentile of all measurements in that sample. The intensity of each clone was then normalized to the median intensity of that clone in the untreated control group. The normalized intensity was then log transformed.

Using Partek Pro™ 5.1, sources of variance, such as batch effects, were identified by Principle Component Analysis (PCA) and appropriate factors were taken into account in the Analysis of Variance (ANOVA). ANOVA was performed to identify the genes that were differentially expressed by cytokine stimulation. Treatment (IL-13 and IL-13R130Q), time (6 hour and 18 hour), and donor (1, 2, and 3) were the three main effects considered in ANOVA. P-value cutoff was 0.05.

Benjamini and Hochberg false discovery rate (FDR) was performed for multiple testing correction. After comparing the gene lists from IL-13 and IL-13R130Q treatments, it was clear that these two treatments resulted in the regulation of the same set of genes. Subsequently, samples from these two treatments were combined and regarded as replicates in ANOVA. Furthermore, outliner samples in the data set were detected by PCA and removed to improve the detection power.

As an alternative approach, fold change comparisons (cutoff = 1.5 fold) between a treated condition and the control were carried out within each donor by using GeneSpring™ 6.2 [18]. A gene was considered as reliably detected in a given condition if more than half of the replicates representing the same condition had a raw expression intensity of more than 50, CV smaller than 25%, and raw intensity being generated from 2 or more of the duplicate spots representing the clone. A pair-wise comparison between a treatment and its untreated control was performed only on the genes that were reliably detected in at least one condition of the pair. The genes that showed at least 1.5 fold differential expression in two or more donors were identified for each cytokine treatment at a time.

### Reverse Transcription and Real Time PCR

1 μg of total RNA from each of the IL-13 (50 ng/ml) or IL-13R130Q (50 ng/ml) or control treated HASMC were used for the reverse transcription (RT) reaction. The RT reaction was performed as per protocol using TaqMan^® ^RT reagents (Applied Biosystems) at 37°C for 120 min followed by 25°C for 10 min. Forty ng of cDNA per reaction were used in the Real Time PCR using the ABI Prism^® ^7900 sequence detection system (Foster City, California). In the presence of AmpliTaq Gold DNA plolymerase (ABI biosystem, Foster City, California), the reaction was incubated for 2 min at 50°C followed by 10 min at 95°C. Then the reaction was run for 40 cycles at 15 sec, 95°C and 1 min, 60°C per cycle. Assays-on-Demand™ primers and probes (Applied Biosystems) were used in the PCR. The Real Time PCR data was analyzed using the standard curve method.

### Flow Cytometry

Flow cytometry was performed as described previously with slight modifications [19]. Briefly, adherent cells treated with IL-13 for 24 hr were washed with PBS, detached by trypsinization (2 min, 37°C) and then washed with Ham's-F12 (10% FCS) media, centrifuged, and transferred to microfuge tubes (1.5 ml). Cells were incubated with anti-IL-13Rα2 (5 μg/ml, Santa Cruz Biotech) antibody followed by 1 hr incubation with a fluorescein isothiocyanate-conjugated secondary antibody (Jackson ImmunoResearch Laboratories; West Grove, PA). In parallel experiements, cells were incubated with the FITC-conjugated mouse anti-VCAM-1 antibody (2 μg/ml, Santa Cruz Biotech) for 1 h at 4°C. The cells were then centrifuged and resuspended in cold PBS in microfuge tubes. Samples were then analyzed using an EPICS XL flow cytometer (Coulter, Hialeah, FL). VCAM-1 and IL-13Rα2 levels were expressed as the increase in mean fluorescence intensity over un-stimulated cells.

## Results

### IL-13 regulates gene expression of HASMCs

IL-13 may exert its deleterious effects in asthma by directly altering gene expression in airway resident cells such as epithelial cells or ASM cells [5-7,20]. In order to determine which genes are regulated by IL-13 in airway smooth muscle cells, we employed the cDNA microarray technology. We also wanted to ascertain if the effect of IL-13R130Q, a naturally occurring isoform of IL-13 and associated with high serum IgE levels [15], was any different than IL-13 in terms of modulating gene expression. The concentrations of IL-13 (10–100 ng/ml) used in our study were shown previously to stimulate gene expression in human ASM cells [7,8,10], although the *in vivo *relevance of these particular concentrations remains unknown.

Three donors were used and two types of analyses were carried out (Fold change analysis; Statistical Analysis). Both IL-13 and IL-13R130Q generated a similar expression profile i.e., genes regulated by IL-13 were the same as those regulated by IL-13R130Q at the 1.5 fold cutoff. Table 2 lists genes of interest that were identified from analyzing the data and divides them into one of three categories. Genes involved in all three characteristics of asthma (airway inflammation, remodeling and bronchial hyper-responsiveness) were identified. Of particular interest are vascular cellular adhesion molecule (VCAM)-1, Tenascin C, IL-13Rα2 and Histamine Receptor H1.

**Table 2:** Summary of genes up regulated by IL-13 and IL-13R130Q.

Category	Gene(s)	Fold change
**Airway Inflammation**		
Adhesion Molecules	**VCAM-1**	↑ **2 fold**
	ALCAM	
	Selectin P ligand	
	Laminin B1	
Chemokines	Chemokine Ligand 2	
	Chemokine Ligand 11	
	Chemokine Ligand 26	
	Chemokine Ligand 27	
Cytokine receptors	**IL-13 Rα2**	↑ **1.6 fold**
	Interleukin 1 receptor	
**Airway Remodeling**		
Extracellular matrix	**Tenascin C**	↑ **2 fold**
	Tenascin R	
	Collagen Type I	
	Collagen Type VI	
	Collagen Type III	
	Fibulin 1	
	CD44	
Cell proliferation	Pim-1	
	eEF1A	
Cytokines	PDGFC	
	Retinoic acid Receptor	
	Interferon beta 1	
**Bronchial Hyper-responsiveness**		
Cytoskeletal constituants	Vimentin	
	Tropomyosin 1	
	Tropomyosin 2	
	Actin	
Calcium regulators	Phospholipase D	
	Calreticulin	
	hGIRK1	
	TRPC4	
	TRPC6	
	Sphingosine kinase 1	
	Rho GDP dissociation inhibitor	
	FKBP1A	
Receptor	**Histamine H1 receptor**	↑ **1.3 fold**

The fold changes correspond to the genes in bold.

### Real Time PCR validation

**Figure 1:**
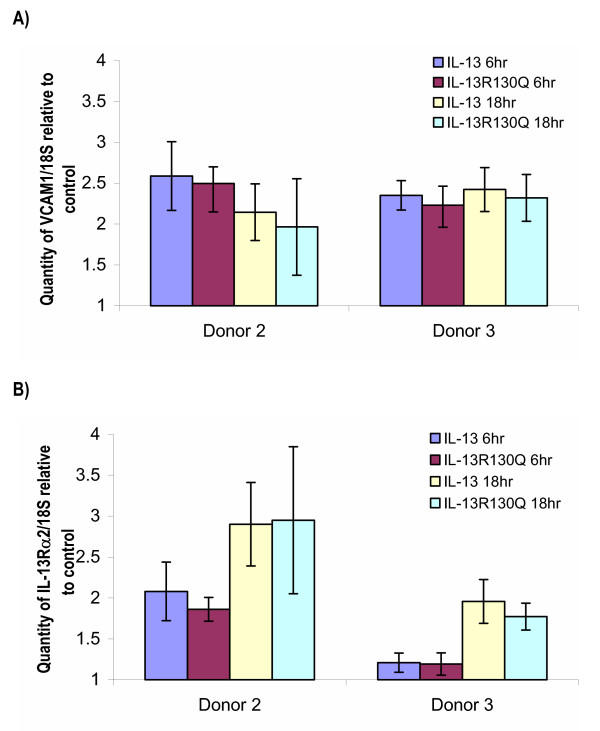
Real Time PCR (Taqman^®^) analysis showing the level of A) VCAM1 B) IL-13Rα2 upon treatment of ASM from two donors with IL-13 or IL-13R13Q for 6 or 18 hrs. The quantity of each gene is normalized to 18S and relative to the untreated sample. Values shown are mean ± standard deviation from an n = 6.

**Figure 2:**
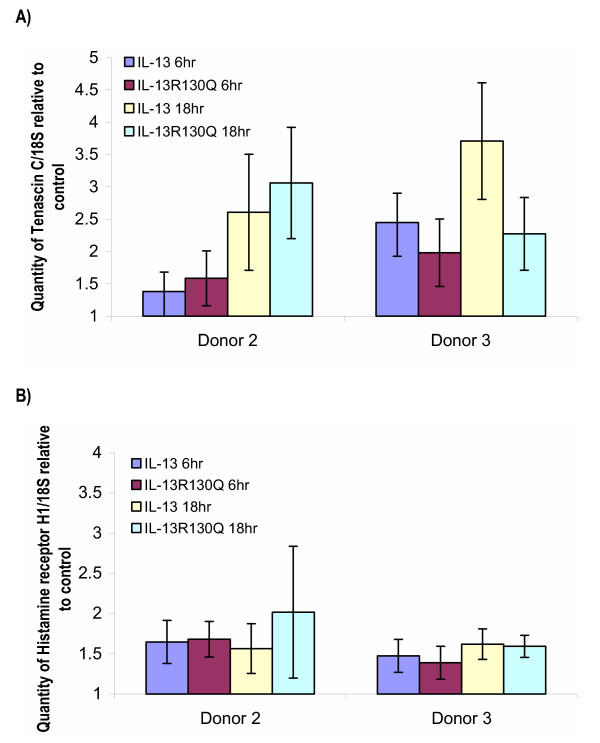
Real Time PCR (Taqman^®^) analysis showing the level of A) Tenascin C and B) Histamine receptor H1 upon treatment of ASM from two donors with IL-13 or IL-13R13Q for 6 or 18 hrs. The quantity of each gene is normalized to 18S and relative to the untreated sample. Values shown are mean ± standard deviation from an n = 6.

TaqMan™ Real Time PCR was used to validate VCAM1, IL-13Rα2, Tenascin C and Histamine Receptor H1. As shown in Figure 1A, VCAM1 was upregulated between 2 and 2.5 fold upon IL-13 or IL-13R130Q treatment at the 6 and 18 hour time points in both donors. This is comparable to the microarray data (Table 2). In Figure 1B, IL-13Rα2 mRNA is upregulated with IL-13 or IL-13R130Q. However, the upregulation is more pronounced at the 18 hour time point compared to 6 hour. In Figure 2A Tenascin C is upregulated with IL-13 and IL-13R130Q and in Figure 2B, Histamine Receptor H1 shows an upregulation of about 1.5 fold in both donors at both time points and with both treatments. Again, this is comparable to the microarray data (Table 2).

### Validation of VCAM-1 and IL-13Rα2 at the protein level

**Figure 3:**
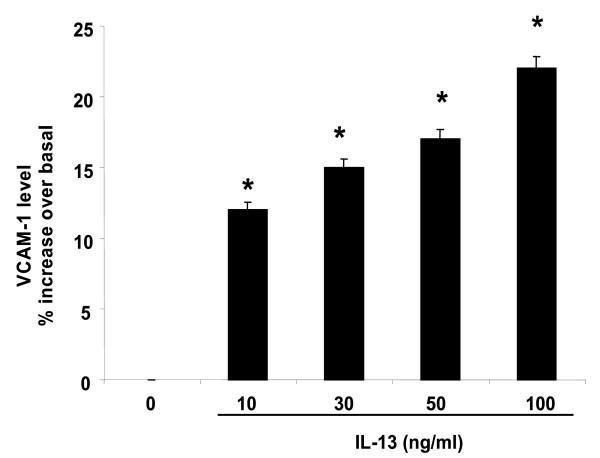
Effect of IL-13 on VCAM-1 expression. ASM cells were incubated with the indicated concentrations of IL-13 for 24 hr. VCAM-1 expression was assessed by flow cytometry as described in Methods. Values shown are mean ± SEM and are significantly different from basal, n = 3 different experiments. *P < 0.05 significant from untreated cell.

**Figure 4:**
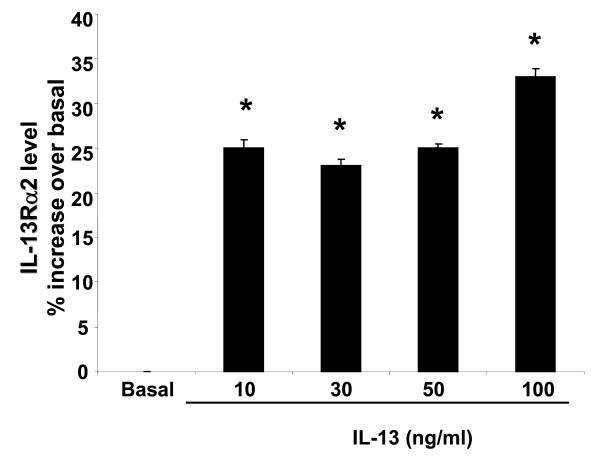
Effect of IL-13 on IL-13Rα2 expression. ASM cells were incubated with the indicated concentrations of IL-13 for 24 hr. IL-13Rα2 expression was assessed by flow cytometry as described in Methods. Values shown are mean ± SEM and are significantly different from basal, n = 3 different experiments. *P < 0.05 significant from untreated cell.

In order to validate the modulatory effect of IL-13 on VCAM-1 and IL-13Rα2 genes at their protein level, flow cytometry was performed to confirm the up regulation of VCAM-1 and IL-13Rα2 in HASMC by IL-13. As shown in Figure 3 and 4, IL-13 (10–100 ng/ml, 24 hr) differentially stimulates the expression of VCAM-1, with levels increasing in a dose-dependent manner, while IL-13Rα2 levels were identical at 10, 30 and 50 ng/ml. At 100 ng/ml IL-13, VCAM-1 and IL-13Rα2 levels were significantly increased by 20% and 35% over basal, respectively (n = 3, p < 0.05). Increases in VCAM-1 and IL-13Rα2 proteins by IL-13 nicely correlate with changes in mRNA levels (Figure 1A and Figure 1B), suggesting that the IL-13 regulates the expression of inflammatory proteins via transcriptional mechanisms.

## Discussion

Recent evidence using various experimental approaches such as gene-deficient mice, soluble inhibitors or transgene overexpressing IL-13 in the airways have highlighted the critical role of IL-13 in the pathogenesis of allergic asthma, possibly due to its ability to regulate goblet cell metaplasia, mucus hypersecretion and airway hyper-responsiveness [1,3,21]. Few reports that used microarray analyses applied to cultured human ASM cells demonstrated that IL-13 differentially regulates a number of important genes that are relevant for the pathogenesis of asthma [7,10]. Although the functional relevance of such gene microarray analyses remains yet uncertain, these studies strongly suggest that IL-13 may be involved in the pathogenesis of asthma by directly modulating physiological responses of the ASM. Compared to the previous gene microarray reports [7,10], we did confirm the physiological relevance of the microarray data using two different experimental approaches. At least for four different genes, VCAM-1 (an adhesion protein), IL-13Rα2, Histamine H1 receptor (a G protein-coupled receptor) and Tenascin C (an extracellular matrix glycoprotein), there was a close correlation between the data obtained from gene microarray with those obtained by real time PCR analyses. In addition, we showed that IL-13 stimulates the expression of VCAM-1 and IL-13Rα2 at the protein level, showing the physiological relevance of the gene array data. It is interesting to note that no difference in gene expression profile were noticeable between cells exposed to IL-13 or IL-13R130Q, an IL-13 polymorphism recently found to be associated with elevated serum and allergen-specific IgE [15,22]. Our report is the first to suggest that this particular IL-13 polymorphism is equally effective as IL-13 in the transcriptional regulation of the genes examined in the present study.

Our present study further supports the concept that IL-13 regulates the expression of different "pro-asthmatic" genes that are potentially important in the regulation of all three key features of asthma, i.e., airway inflammation, airway remodeling and bronchial hyper-responsiveness (for details see Table 2). Previous reports using cultured ASM cells also demonstrated that IL-13 can stimulate the expression of other pro-inflammatory proteins, such as eotaxin [8,23,24], TARC [9] or VEGF [25] and tenascin [present report and [10]]. Upregulation of tenascin C and R, glycoproteins that contribute to extracellular matrix structure [26], may play an important role in airway remodeling, a characteristic of chronic asthma. The stimulatory role of IL-13 on VCAM-1 may be important in asthma since VCAM-1 has been regarded as a key player in the development of airway inflammation [27]. The ability of IL-13 to increase the expression of different G-protein coupled receptors (GPCR), such as Histamine H1 [present report and [10]] or CysLT1 receptor [28] represents one potential mechanism by which IL-13 promotes airway hyper-responsiveness to GPCR agonists previously described both *in vivo *[1,3,21] or *in vitro *in isolated airways preparations [11,29,30]. Additional studies are needed to determine whether IL-13 also modulates ASM responsiveness to Histamine.

The receptor complex by which IL-13 regulates cellular function comprises the IL-13Rα1, which binds IL-13 and forms a complex with the IL-4Rα to initiate signal transduction via the JAK/STAT6 pathway [31]. IL-13Rα2, the other cell surface protein binds IL-13 with high affinity but the complex is not functionally active. One previous report using transgenic mice showed that overexpressing IL-13 in the airways induced a marked increase in both IL-13Rα1 and IL-13Rα2 mRNA levels, mostly in epithelial cells and macrophages [32]. Our study is the first to show that the expression of IL-13Rα2 is also transcriptionally increased by IL-13 in ASM cells. Although the functional significance of such regulation remains unknown, it is possible that the newly induced IL-13Rα2 could function as a decoy receptor to limit IL-13 signaling in ASM cells. Additional studies are needed to further support this hypothesis.

## Conclusions

These data further support the hypothesis that gene modulation by IL-13 in ASM may be essential for the events leading to the development of allergic asthma. Additional studies are clearly needed to define the transcriptional regulation of the different "pro-asthmatic" genes by IL-13, which may lead to novel therapeutic approaches for the treatment of allergic asthma.

## Authors' contributions

FS, LL, YA and RAP participated in the conception and design of the study. FS coordinated the study and along with YA drafted the manuscript. OT performed the RNA isolation and flow cytometry experiments. CH and KL performed the microarray data analysis. MB performed the Real Time PCR experiments. DG and BA proof read the manuscript. All authors read and approved the final manuscript.
